# The Influence of Smoking and Co-morbidity on Dose Achievement in Primary or Adjuvant Radio(Chemo)Therapy in Head and Neck Squamous Cell Carcinoma (HNSCC)

**DOI:** 10.3389/fonc.2020.00398

**Published:** 2020-03-24

**Authors:** Asita Fazel, Elgar Susanne Quabius, Alexander Fabian, Thilo Schleicher, Konstantin Kress, Martin Laudien, Karen Huber, Arved Herzog, Mireia Gonzales Donate, Markus Hoffmann

**Affiliations:** ^1^Department of Otorhinolaryngology, Head and Neck Surgery, Christian-Albrechts-University Kiel, Kiel, Germany; ^2^Institute of Immunology, Christian-Albrechts-University Kiel, Kiel, Germany; ^3^Department of Radiation Oncology, Christian-Albrechts-University Kiel, Kiel, Germany

**Keywords:** co-morbidity, therapy compliance, dose achievement, smoking, radiochemotherapy, radiotherapy

## Abstract

**Introduction:** Smoking has a negative impact on survival of HNSCC patients. In addition, smoking is associated with the prevalence of co-morbidities and, thus, it may be assumed that not smoking *per se* but co-morbidities impact the course of therapy in terms of lower compliance and dose-reduction. However, data addressing this issue is sparse and conflicting at present, specifically for HNSCCs.

**Patients and methods:** Patient files and tumor documentation from 643 consecutive cases of the University Head and Neck Cancer Centre Kiel were analyzed retrospectively. Patient characteristics and smoking habits were assessed and correlated with co-morbidities and course of treatment.

**Results:** The examined 643 patient files showed that 113 (17.6%), 349 (54.3%), and 180 (28%) patients were never, active, and former smokers, respectively. Three hundred fifteen (49%) were treated by surgery only; 121 (18.8%) received surgery + adjuvant RCT and 72 (11.2%) surgery + adjuvant RT. 111 (17.3%) received primary RCT and 24 (3.7%) primary RT. 131 (20.4%) and 512 (79.6%) had no or had co-morbidities, respectively. Smoking (>10 py) was significantly associated with co-morbidities (*p* = 0.002). However, smoking and co-morbidities, neither alone nor in combination, were correlated with failure in reaching target doses of radio(chemo)therapy (*p* > 0.05). Applying (verified) Carlson-Comorbidity-Index (CCI) did not change the results.

**Conclusions:** As expected, smoking is significantly associated with co-morbidities. Dose-reduction of radio(chemo)therapy is as common among active smokers and patients with co-morbidities as among never smokers and patients without co-morbidities. Thus, smoking and co-morbidity seems to impact survival by other means than impairing planned therapy regimens.

## Introduction

Head and neck squamous cell carcinoma (HNSCC) represents with 7% of all malignant diseases the 6th most common malignancy in humans and accounts for ~70,000 new cases diagnosed and ~10,000 deaths annually in the USA and Europe (1, 2). Carcinogenic factors for HNSCCs are the ingredients of tobacco smoke and alcohol (3) and, specifically for tonsillar SCC (TSCC), the infection with high-risk human papillomaviruses [HPV] (4, 5). Moreover, an increasing prevalence of HPV-positive TSCC seems to be paralleled by a decreased tobacco use (6). Even though in our own study populations the proportion of smokers among HPV-negative and HPV-positive TSCC patients is 70 and 50%, respectively (5), there is a clear tendency of HPV-positive HNSCC patients to be non-smokers and vice versa in various populations tested throughout the world [(4–7) and references cited therein]. Therapy regimens for HNSCCs are surgery alone or combined with risk adapted adjuvant radio(chemo)therapy (R(C)T) and primary R(C)T. Prognosis of survival is predominantly dependent on the infection with HPV (4, 5, 8), smoking habit (9) and the presence of co-morbidities (10) with poorest survival rates for HPV-negative smokers or patients with co-morbidities (4, 5, 8–11). Only recently, we reported on inferior survival rates even for HPV-positive HNSCCs cases when the patients were former or active smokers (5). Since it is established that a positive smoking history is significantly linked to the presence of co-morbidities (11–13) it could be assumed that not the smoking habit *per se* is causally linked to poor survival rates of smokers but rather to smoking associated co-morbidities of these patients (14). Smoking induced co-morbidity might therefore affect therapy compliance and regimens for example in terms of not reaching planned dosage of radiation or chemotherapy. While the specific link of smoking and co-morbidity has not yet been elucidated in this setting, Chen et al. reported an extended duration of radiotherapy for nasopharyngeal carcinoma patients in the presence of co-morbidity itself (15). Furthermore, Lee et al. described inferior tolerability and use of chemotherapy for solid tumors in co-morbid patients (16). On the contrary, Bøje et al. report a similar therapy compliance of HNSCC patients with or without co-morbidity (17). In this light of conflicting data, we aimed to investigate the influence of co-morbidity on therapy compliance of HNSCC patients with an emphasis on their smoking history. For this purpose, we analyzed patient files and data retrieved from our hospital database of 643 patients treated in an academic Head and Neck Cancer Center in Germany.

## Patients and Methods

### Study Design

Data were extracted from a clinical database and files of 643 consecutive unselected cancer patients, who were treated at the University Hospital Head and Neck Tumor Center Kiel, Germany, between 2013 and 2016 were analyzed in a retrospective setting. Overall, patient files of all patients with curative intent treated in the aforementioned time period were analyzed. This included patients with all types of HNSCC and all TNM-categories. M1 patients were also treated with primary radiochemotherapy, possibly after resection of distant metastasis. Study design and work-up followed informed and written consent; the study was approved by the local Ethics Committee (D507/17).

The aim of the study was to assess based on the patients' files whether or not the patients reached the optimal target dose of 300 mg Cisplatin or Carboplatin per m^2^ body surface area (BSA) and to establish a possible correlation between smoking habit and co-morbidities of the patients, alone and in combination, on therapy compliance (reaching the optimal target dose). Since it is well known that 200 mg Cisplatin/m^2^ BSA, is also sufficient for the here applied concomitant chemotherapy, a second analysis was performed based on this lower, but still sufficient dose, again correlating therapy compliance or the lack of it with smoking habit and co-morbidities of the patients, alone and in combination. Similarly, two different cut-off levels were used to correlate achievement of the target dose for primary and adjuvant radiotherapy (RT), respectively, with smoking habit and co-morbidities of the patients, alone and in combination. These doses are: 70 and 65 gy for primary RT and 60 and 55 gy for adjuvant RT.

Smoking habit was measured in pack years consumed and patients who stopped smoking at least 2 years prior to diagnosis were classified as former smokers. In order to assess and screen the co-morbidity burden comprehensively, we used four different algorithms: The different algorithms are characterized as follows: [1] “counted”: the number of all co-morbidities (listed and not listed in CCI) of each patient was counted, added up and each co-morbidity was scored as “1”; [2] CCI: the co-morbidities were scored according to Charlson et al. (18); [3]: “CCI plus” presents an “in-house” modification of the original CCI, where arterial hypertension and arrhythmia are included and both are scored as “1”; [4]: the Quan CCI was used as described, previously (19).

In addition, overall survival (OS) and tumor related survival (TRS) with a follow-up range of 0.01–4.4 years and mean follow up of 1.62 years were analyzed. OS was defined as time in years from date of diagnosis until date of death from either any cause or primary tumor related death (TRS). Mortality was censored on October 1st, 2017 and Kaplan–Meier analysis were performed correlating the effect of smoking and co-morbidity alone and in combination on overall and tumor related survival. Furthermore, to describe the patient cohort in more detail, the following information was also extracted from the patients' files: sex, smoking habit prior to diagnosis, status (censored 10.2017), cause of death, tumor site, T- N- M-categories, treatment, occurrence of co-morbidities and their entities.

Analysis regarding the correlation of smoking habit and occurrence of co-morbidities and in the respective survival analysis included patients receiving “surgery only” to strengthen statistical power of analysis. However, these patients were not included in the data set used to analyze therapy compliance.

### Statistical Analyses

Fisher's exact test (SPSS 20.0 software) was performed to correlate co-morbidity and smoking either alone or in combination with the patients' therapy compliance. One way ANOVA (SPSS 20.0 software) was performed to correlate patients' age and the occurrence of co-morbidities and the patients' tobacco consumption with the occurrence of co-morbidities. In addition Kaplan–Meier plots and log-rank tests (SPSS 20.0 software) were used correlating the effect of smoking and co-morbidity alone and in combination on overall and tumor related survival. *p*-values ≤ 0.05 were considered statistically significant.

## Results

### Patient Demographics

Patient characteristics are described in Table 1. The mean age was 64.5 ± 10.2 years (range 39.86–98.02 years). The majority of patients was male [77.3% (497/643)]. In total, 349 patients (54.4%) reported a smoking habit, 113 (17.6%) reported to have never smoked and 180 (28.0%) reported to have ceased smoking at least 2 years prior to diagnosis. Tumor sites were as follows: oral cavity 51 patients (7.9%); hypopharynx 94 patients (14.6%); larynx 163 patients (25.3%); tonsil 95 patients (14.8%); oropharynx other than tonsil 155 patients (24.1%); nasopharynx 49 patients (7.6%) and other sites 36 patients (5.6%). The majority of patients were treated by surgery only (315/643; 49.0%); 111 patients (17.3%) were treated by primary radiochemotherapy (RCT); 24 patients (3.7%) received primary radiotherapy (RT); 121 patients (18.8%) were treated by surgery plus adjuvant RCT, and 72 patients (11.2%) were treated by surgery plus adjuvant RT. Of those patients treated by primary or adjuvant RCT (*n* = 232) 59.5% (*n* = 137) were treated with cisplatin, for 22 of these patients no cisplatin dose was recorded. Of the remaining 115 patients with known cisplatin dose 65 patients (56.5%) did not reach the target dose of 300 mg/m^2^ body surface area (BSA) and 50 patients (43.5%) did reach this target dose. However, 95 patients (82.6%) did reach the reduced, but still sufficient target dose of 200 mg/m^2^ BSA. In contrast to the cisplatin data radiotherapy data were available for all 137 patients treated with cisplatin as part of their RCT. Of these 137 patients only 22.4% (*n* = 15) patients treated by primary RCT reached the target dose of 70 gy, the dose of 65 gy, however, was reached by 89.6% (*n* = 60) of the patients. Of those patients treated by adjuvant RCT 82.9% (*n* = 58) reached the target dose of 60 gy and all 70 patients reached the lower dose of 55 gy. Of those patients treated by primary RT without chemotherapy, only 20.8% (*n* = 5) reached the target dose of 70 gy. Additionally, in this treatment group 58.3% (*n* = 14) of the patients reached 65 gy. In the group of patients treated with adjuvant RT without chemotherapy 47/71 patients (66.2%) reached 60 gy and 63 (88.7%) reached 55 gy.

**Table 1:** Patient characteristics.

**Variable**	***n***	**percent**
Sex		
Female	146	22.7
Male	497	77.3
Smoking habit prior to diagnosis (*n* = 1 missing)		
Never smoker	113	17.6
Former smoker	180	28.0
Active smoker	349	54.4
Status (10.2017)		
Alive	501	77.9
Dead	142	22.1
Cause of death		
Primary tumor	87	61.3
Secondary tumor	6	4.2
Not tumor related	23	16.2
Unclear	26	18.3
Tumor site		
Oral cavity	51	7.9
Hypopharynx	94	14.6
Larynx	163	25.3
Tonsil	95	14.8
Oropharynx other than tonsil	155	24.1
Nasopharynx	49	7.6
Other	36	5.6
T-category (*n* = 7 missing)		
T1/T2	318	50.0
T3/T4	318	50.0
N-category (*n* = 4 missing)		
N0	318	50.0
N1-2a	292	45.9
>N2b	29	4.6
M-category (*n* = 45 missing)		
M0	580	91.2
M1	18	2.8
Treatment		
Surgery only	315	49.0
Adjuvant RCT	121	18.8
Adjuvant RT	72	11.2
Primary RCT	111	17.3
Primary RT	24	3.7
Co-morbidities						
Minus	131	20.4
Plus	512	79.6
Co-morbidity entity (*n* = 1,152 since several patients have more than one co-morbidity)		
Cardiovascular	585	50.8
Of these		
Cardial	52	8.9
Vascular	263	45.0
Cardiovascular	270	46.2
Pulmonary	124	10.8
Endocrinological	207	18.0
Other	236	20.5

One hundred thirty-one (20.4%) patients only had no further disease than the head and neck tumor, while the majority of patients (*n* = 512; 79.6%) were treated for one or more further diseases. The total number of reported co-morbidities was 1,152, of these cardio-vascular diseases presented with 585 reports (50.8%) the majority of co-morbidities. Pulmonary diseases were reported 124 times (10.8%), endocrinological onces 207 times (18.0%) and other co-morbidities were reported 236 times (20.5%).

### Comparison of Different Algorithms to Quantify Co-morbidities

In Table 2 the scoring results for the reported co-morbidities are shown. For better comparison the patient number (*n* = 643) was set as 100% since different algorithms score the same co-morbidities differently. The different algorithms revealed no significant differences in the observed co-morbidity scores (*p* = 1). All further analysis regarding the effects of the patients' co-morbidities was performed using the “counted” index, since this is the only algorithm including all co-morbidities, hence presenting the least bias.

**Table 2:** Comparison of different algorithms to quantify co-morbidities.

**Algorithm**	**Counted**		**CCI**		**CCI plus**		**Quan CCI**	
**Comorbidities**	***n***	**%**	***n***	**%**	***n***	**%**	***n***	**%**
0	131	20.4	285	44.3	180	28.0	391	60.8
1–3	433	67.3	298	46.3	363	56.5	210	32.7
>4	79	12.3	60	9.3	100	15.6	42	6.5

For better comparison the patient number (n = 643) was set as 100% since different algorithms score the same co-morbidities differently. The different algorithms are characterized as follows: counted: the number of all co-morbidities of each patient was counted, added up and each co-morbidity was scored as “1”; for Charlson co-morbidity index (CCI) the co-morbidities were scored according to Charlson et al. (18). CCI plus presents an “in-house” modification of the CCI, where arterial hypertension and arrhythmia are included in the original CCI and both are scored as “1.” The Quan CCI was used as described, previously. Here co-morbidities are pooled and grouped into none, 1–3 and more than four co-morbidities (19).

*Kruskal-Wallis Test: p = 1*.

### Analysis of Age, Smoking Habit and Co-morbidity

**Figure 1:**
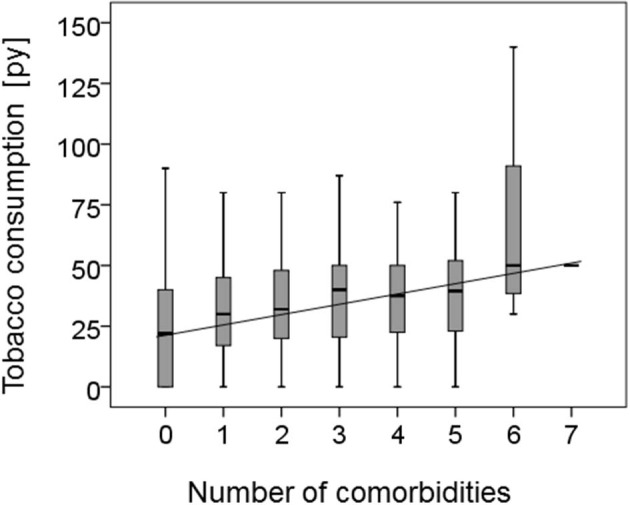
Correlation of smoking habit and co-morbidity. The correlation between the reported number of co-morbidities of the HNSCC patients and their tobacco consumption in pack years (py) is shown as a boxplot. In addition the regression co-efficiency was calculated demonstrating a correlation (*r*^2^ = 0.031) and an overall significance calculated by one way ANOVA of *p* = 0.046 between tobacco consumption and increasing number of co-morbidities.

Patients reporting to be active smokers were at time of cancer diagnosis significantly younger than never or former smokers (never smokers: 68.6 ± 10.9; former smoker: 68.4 ± 9.7; active smoker: 61.0 ± 8.5; *p* < 0.001). In addition, active smokers had a total of 611 co-morbidities, former smokers had 391 and never smokers 146 co-morbidities (*p* = 0.030); for one patient with four co-morbidities the smoking habit was not recorded. Further analysis of the patients' age with their smoking habit in combination with the occurrence of co-morbidities showed that never smokers, former smokers and active smokers without co-morbidities were significantly younger than never smokers, former smokers and active smokers with co-morbidities overall *p*-value by one way ANOVA was *p* < 0.0001. Tukey-Kramer Multiple *post hoc* comparison test showed: never smokers with co-morbidities: 70.9 ± 10.2; never smokers without co-morbidities: 63.4 ± 10.9 *p* < 0.01; former smokers with co-morbidities: 69.2 ± 9.2 former smokers without co-morbidities: 63.1 ± 10.9 *p* < 0.05; active smokers with co-morbidities 62.1 ± 8.9 active smokers without co-morbidities 56.9 ± 7.5 *p* < 0.001. In addition, as shown in Figure 1 there is a correlation (*r*^2^ = 0.031) between tobacco consumption in pack years and the number of co-morbidities reported together with an overall significance (one way ANOVA) of *p* = 0.046 between tobacco consumption and number of co-morbidities.

### Effect of Smoking and Co-morbidity Alone and in Combination on Overall and Tumor Related Survival

**Figure 2:**
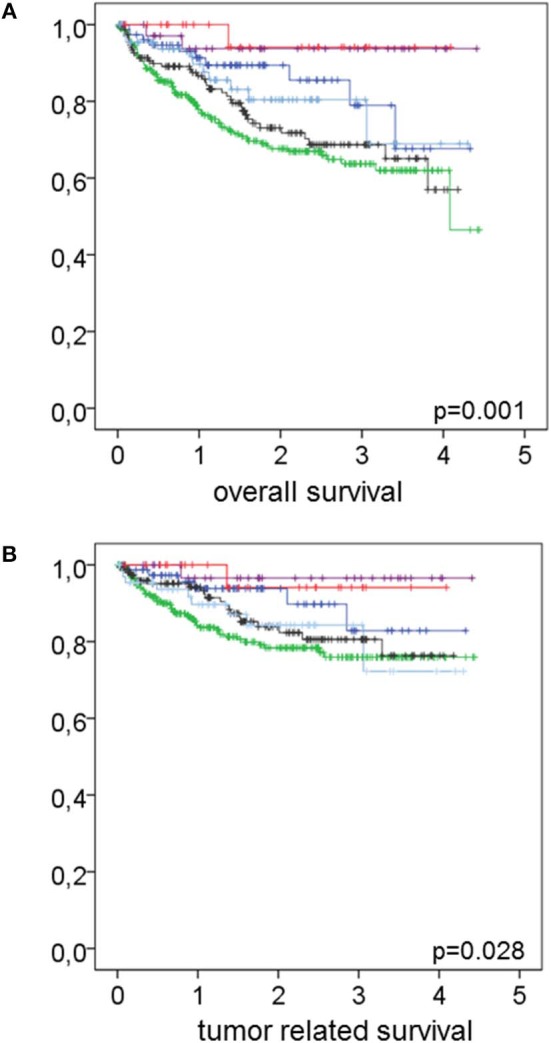
Effect of the combination of smoking habit and co-morbidity on overall and tumor related survival. In **(A)** overall and in **(B)** tumor related survival in relation to the combination of smoking habit and co-morbidity is shown. Never smokers without co-morbidities (magenta lines) *n* = 38 had an overall survival rate of 93.7% and a tumor related survival rate of 96.6%. Former smokers without co-morbidities (red lines) *n* = 26 had overall and tumor related survival rates of 94.1% each. Active smokers without co-morbidities (light blue lines) *n* = 67 had an overall survival rate of 80.4% and a tumor related survival rate of 84.3%. Never smokers with co-morbidities (dark blue lines) *n* = 78 had an overall survival rate of 78.9% and a tumor related survival rate of 82.8%. Former smokers with co-morbidities (black lines) *n* = 153 had an overall survival rate of 68.7% and a tumor related survival rate of 80.6%. Active smokers with co-morbidities (green lines) *n* = 280 had an overall survival rate of 63.7% and a tumor related survival rate of 75.9%. Log rank test revealed *p* = 0.001 and 0.028 for overall and tumor related survival, respectively.

Table 3 shows the influence of smoking habit, the occurrence of co-morbidities either alone or in combination on the patients' overall or tumor related survival. Never smokers showed better overall and tumor related survival than former and active smokers (*p* = 0.002 and 0.006 for overall and tumor related survival, respectively). Likewise, patients without co-morbidities showed better overall and tumor related survival than patients with co-morbidities (*p* = 0.001 and 0.046 for overall and tumor related survival, respectively). Interestingly, as also shown in Figures 2A,B, the combination of smoking habit and occurrence of co-morbidities revealed that never smokers with co-morbidities showed worse overall and tumor related survival than former and even active smokers without co-morbidities: 3 years overall survival was 78.9% for never smokers with co-morbidities and 94.1% for former, and 80.4% for active smokers without co-morbidities; 3 years tumor related survival was 82.8% for never smokers with co-morbidities and 94.1% for former and 84.3% for active smokers without co-morbidities.

**Table 3:** Kaplan-Meyer analysis showing overall (OS) and tumor related (TRS) survival dependent on smoking habit, patients' comorbidity and the correlation of both.

**Variable**	***n***	**Mean (years)**	**Std (years)**	**Min (years)**	**Max (years)**	**Median (years)**	**3ry OS (%)**	***p*-value**	**3yr TRS (%)**	***p*-value**
**Smoking habit prior to diagnosis**										
Never smoker	113	1.72	1.18	0.02	4.41	1.47	85.7	0.002	89.7	0.006
Former smoker	180	1.69	1.14	0.02	4.18	1.54	72.3		82.6	
Active smoker	349	1.55	1.14	0.01	4.44	1.26	66.3		76.9	
**Comorbidity**										
Minus	131	1.74	1.17	0.02	4.41	1.61	87.0	0.001	89.9	0.046
Plus	512	1.59	1.14	0.01	4.44	1.38	67.4		78.4	
**Smoking habit prior to diagnosis in combination with co-morbidity**										
Never smoker without co-morbidity	38	1.97	1.36	0.03	4.41	1.76	93.7	0.001	96.6	0.028
Former smoker without co-morbidity	26	1.91	1.07	0.36	4.09	1.62	94.1		94.1	
Active smoker without co-morbidity	67	1.49	1.05	0.02	4.30	1.39	80.4		84.3	
Never smoker with co-morbidity	78	1.59	1.07	0.02	4.33	1.39	78.9		82.8	
Former smoker with co-morbidity	153	1.65	1.15	0.02	4.18	1.53	68.7		80.6	
Active smoker with co-morbidity	280	1.56	1.15	0.01	4.44	1.24	63.7		75.9	

### Patients' Therapy Compliance in Relation to Smoking Habit and Co-morbidity Alone and in Combination

The effects of smoking habit and occurrence of co-morbidities alone and in combination on the patients' therapy compliance are shown in Table 4. As shown in Table 4A, patients' therapy compliance was initially defined as reaching the target dose of 300 or 200 mg cisplatin/m^2^ body surface area (BSA). Of the 232 patients treated by RCT 137 (59.5%) were treated with cisplatin. For 115 (83.3%) of these patients the cisplatin dose could be retrieved form the patient files. The target dose of 300 mg cisplatin/m^2^ BSA was reached by 50 (43.5%) of the patients, while 65 (56.5%) of the patients did not reach this target dose. The reduced, but still sufficient target dose of 200 mg cisplatin/m^2^ BSA was reached by 95 (82.6%) of the patients while 20 (17.4%) of the patients did also not reach this reduced target dose. However, there was no correlation between smoking habit, the occurrence or the number of co-morbidities and whether or not any of the target doses were reached. Nor had the combination of smoking habit and the occurrence of co-morbidities any effect on therapy compliance regarding the cisplatin dose. While the cisplatin target dose is identical during primary and adjuvant RCT the target gray dose for primary R(C)T is 70 gy or, as a second option, 65 gy, and for adjuvant R(C)T the target dose is either 60 or 55 gy. For this reason, in Tables 4B,C target doses for the RT part of the RCT (Table 4B) and the RT alone (Table 4C) are divided in primary and adjuvant RT. Only 22.4% (*n* = 15) patients treated by primary RCT reached the target dose of 70 gy, the dose of 65 gy, however, was reached by 89.6% (*n* = 60) of the patients. Of those patients treated by adjuvant RCT 82.9% (*n* = 58) reached the target dose of 60 gy and all 70 patients reached the lower dose of 55 gy. Again, no correlation between smoking habit, the occurrence or the number of co-morbidities and whether or not any of the target doses were reached could be detected. Nor had the combination of smoking habit and the occurrence of co-morbidities any effect on therapy compliance regarding the RT part of the RCT.

**Table 4A:** Correlation between therapy compliance during R(C)T treatment and the patients' smoking habit, the occurrence of co-morbidities and the combination of both parameters.

**A: Reaching the cisplatin target dose during RCT treatment. Of the 232 patients treated by primary or adjuvant RCT 137 were treated with cisplatin, however, for 22 patients the cisplatin dose was not recorded**.						
	**Cisplatin target dose 300 mg/BSA**			**Cisplatin target dose 200 mg/BSA**		
	***n*** **=** **115**			***n*** **=** **115**		
	**Reached**			**Reached**		
	Yes	No		Yes	No	
Variable	50 (43.5)	65 (56.5)		95 (82.6)	20 (17.4)	
**Smoking habit prior to diagnosis**			*p*-value			*p*-value
Never smoker	3 (2.6)	13 (11.3)	0.101	12 (10.4)	4 (3.5)	0.408
Former smoker	13 (11.3)	16 (13.9)		26 (22.6)	3 (2.6)	
Active smoker	34 (29.6)	36 (31.3)		57 (49.6)	13 (11.3)	
Co-morbidities						
Minus	10 (8.7)	14 (12.2)	1	20 (17.4)	4 (3.5)	0.408
Plus	40 (34.8)	51 (44.3)		75 (65.2)	16 (13.9)	
**Co-morbidities**						
None	10 (8.7)	14 (12.2)	1	20 (17.4)	4 (3.5)	1
1–3 co-morbidities	36 (31.3)	46 (40.0)		68 (59.1)	14 (12.2)	
>4 co-morbidities	4 (3.5)	5 (4.3)		7 (6.1)	2 (1.7)	
**Smoking habit in combination with co-morbidities**						
Never smoker without co-morbidity	1 (0.9)	5 (4.3)	0.457	4 (3.5)	2 (1.7)	0.758
Former smoker without co-morbidity	2 (1.7)	2 (1.7)		4 (3.5)	0 (0.0)	
Active smoker without co-morbidity	7 (6.1)	7 (6.1)		12 (10.4)	2 (1.7)	
Never smoker with co-morbidity	2 (1.7)	8 (7.0)		8 (7.0)	2 (1.7)	
Former smoker with co-morbidity	11 (9.6)	14 (12.2)		22 (19.1)	3 (2.6)	
Active smoker with co-morbidity	27 (23.5)	29 (25.2)		45 (39.2)	11 (9.6)	

*Numbers in parentheses represent percentages. BSA, body surface area*.

**Table 4B:** 

**B: Reaching the target dose of either primary or adjuvant RT during RCT treatment. In contrast to the cisplatin data radiotherapy data were available for all 137 patients**												
	**Primary RT target dose 70 gy**			**Primary RT target dose 65 gy**			**Adjuvant RT target dose 60 gy**			**Adjuvant RT target dose 55 gy**		
	***n*** **=** **67**			***n*** **=** **67**			***n*** **=** **70**			***n*** **=** **70**		
	**Reached**			**Reached**			**Reached**			**Reached**		
	Yes	No		Yes	No		Yes	No		Yes	No	
Variable	15 (22.4)	52 (77.6)		60 (89.6)	7 (10.4)		58 (82.9)	12 (17.1)		70 (100.0)	0 (0.0)	
**Smoking habit prior to diagnosis**		*p*-value			*p*-value			*p*-value			*p*-value	
Never smoker	0 (0.0)	10 (15.0)	0.127	9 (13.5)	1 (1.5)	0.853	9 (12.9)	2 (2.9)	0.464	11 (15.7)		NA
Former smoker	5 (7.5)	10 (15.0)		13 (19.5)	2 (3.0)		21 (29.9)	2 (2.9)		23 (32.9)		
Active smoker	10 (15.0)	32 (48.0)		38 (56.5)	4 (6.0)		28 (39.8)	8 (11.6)		36 (51.4)		
**Co-morbidities**												
Minus	2 (3.0)	10 (15.0)	0.721	9 (13.5)	3 (4.5)	0.336	18 (25.7)	3 (4.3)	1	21 (29.9)		NA
Plus	13 (19.4)	42 (62.7)		51 (76.0)	4 (6.0)		40 (57.1)	9 (12.9)		49 (70.1)		
**Co-morbidities**												
None	2 (3.0)	10 (15.0)	0.370	9 (13.5)	3 (4.5)	0.192	18 (25.7)	3 (4.3)	0.879	21 (29.9)		NA
1–3 co-morbidities	10 (15.0)	38 (57.0)		44 (66.0)	4 (6.0)		36 (51.4)	9 (12.9)		45 (64.4)		
>4 co-morbidities	3 (4.5)	4 (6.0)		7 (10.5)	0 (0.0)		4 (5.7)	0 (0.0)		4 (5.7)		
**Smoking habit in combination with co-morbidities**												
Never smoker without co-morbidity	0 (0.0)	3 (4.5)	0.373	2 (3.0)	1 (1.5)	0.079	4 (5.7)	1 (1.4)	0.884	5 (7.1)		NA
Former smoker without co-morbidity	1 (1.5)	3 (4.5)		2 (3.0)	2 (3.0)		5 (7.1)	0 (0.0)		5 (7.1)		
Active smoker without co-morbidity	1 (1.5)	4 (6.0)		5 (7.5)	0 (0.0)		9 (12.9)	2 (2.9)		11 (15.7)		
Never smoker with co-morbidity	0 (0.0)	7 (10.5)		7 (10.5)	0 (0.0)		5 (7.1)	1 (1.4)		6 (8.6)		
Former smoker with co-morbidity	4 (6.0)	7 (10.5)		11 (16.5)	0 (0.0)		16 (22.9)	2 (2.9)		18 (25.7)		
Active smoker with co-morbidity	9 (13.5)	28 (42.0)		33 (49.5)	4 (6.0)		19 (27.1)	6 (8.6)		335 (35.7)		

*Numbers in parentheses represent percentages; NA, not applicable; since all patients reached the target dose no statistical analysis could be performed*.

**Table 4C:** 

**C: Reaching the target dose during either primary or adjuvant RT**												
	**Primary RT target dose 70 gy**			**Primary RT target dose 65 gy**			**Adjuvant RT target dose 60 gy**			**Adjuvant RT target dose 55 gy**		
	***n*** **=** **24**			***n*** **=** **24**			***n*** **=** 71*			***n*** **=** 71*		
	**Reached**			**Reached**			**Reached**			**Reached**		
	Yes	No		Yes	No		Yes	No		Yes	No	
Variable	5 (20.8)	19 (79.2)		14 (58.3)	10 (41.7)		47 (66.2)	24 (33.8)		63 (88.7)	8 (11.3)	
**Smoking habit prior to diagnosis**			*p*-value			*p*-value			*p*-value			*p*-value
Never smoker	0 (0.0)	5 (20.8)	0.656	4 (16.7)	1 (4.2)	0.498	10 (14.1)	9 (12.7)	0.092	16 (22.5)	3 (4.2)	0.431
Former smoker	2 (8.3)	5 (20.8)		4 (16.7)	3 (12.5)		11 (15.5)	8 (11.3)		16 (22.5)	3 (4.2)	
Active smoker	3 (12.5)	9 (37.5)		6 (25.0)	6 (25.0)		26 (36.6)	7 (9.9)		31 (43.7)	2 (2.8)	
**Co-morbidities**												
Minus	0 (0.0)	0 (0.0)	NA	0 (0.0)	0 (0.0)	NA	11 (15.5)	4 (5.6)	0.759	13 (18.3)	2 (2.8)	0.673
Plus	5 (20.8)	19 (79.2)		14 (58.3)	10 (41.7)		36 (50.7)	20 (28.2)		50 (70.4)	6 (8.5)	
**Co-morbidities**												
None	0 (0.0)	0 (0.0)	1**	0 (0.0)	0 (0.0)	0.55**	11 (15.4)	4 (5.6)	0.682	13 (18.3)	2 (2.8)	0.726
1–3 co-morbidities	5 (20.8)	16 (66.7)		13 (54.2)	8 (33.3)		30 (42.3)	18 (25.4)		42 (59.1)	6 (8.5)	
>4 co-morbidities	0 (0.0)	3 (12.5)		1 (4.2)	2 (8.3)		6 (8.5)	2 (2.8)		8 (11.3)	0 (0.0)	
**Smoking habit in combination with co-morbidities**												
Never smoker without co-morbidity	0 (0.0)	0 (0.0)	0.656**	0 (0.0)	0 (0.0)	0.498**	3 (4.2)	1 (1.4)	0.207	3 (4.2)	1 (1.4)	0.375
Former smoker without co-morbidity	0 (0.0)	0 (0.0)		0 (0.0)	0 (0.0)		3 (4.2)	1 (1.4)		4 (5.6)	0 (0.0)	
Active smoker without co-morbidity	0 (0.0)	0 (0.0)		0 (0.0)	0 (0.0)		5 (7.0)	2 (2.8)		6 (8.4)	1 (1.4)	
Never smoker with co-morbidity	0 (0.0)	5 (20.8)		4 (16.7)	1 (4.2)		7 (9.9)	9 (12.7)		14 (19.8)	2 (2.8)	
Former smoker with co-morbidity	2 (8.3)	5 (20.8)		4 (16.7)	3 (12.5)		8 (11.3)	6 (8.5)		11 (15.6)	3 (4.2)	
Active smoker with co-morbidity	3 (12.5)	9 (37.5)		6 (25.0)	6 (25.0)		21 (29.6)	5 (7.0)		25 (35.2)	1 (1.4)	

Numbers in parentheses represent percentages; *For 1 patient treated by adjuvant RT smoking habit was not recorded; NA, not applicable; since there were no patients that had no co-morbidities no statistical analysis could be performed; **patients without co-morbidities were not included into the analysis.

Similar as seen for the RT part of the RCT regimen, of those patients treated by primary RT without chemotherapy, only 20.8% (*n* = 5) reached the target dose of 70 gy. Additionally, in this treatment group only 58.3% (*n* = 14) of the patients reached 65 gy. In the group of patients treated with adjuvant RT without chemotherapy 47/71 patients (66.2%) reached 60 gy and 63 (88.7%) reached 55 gy. Here again no correlation between smoking habit, the occurrence or the number of co-morbidities and whether or not any of the target doses were reached could be detected. Nor had the combination of smoking habit and the occurrence of co-morbidities any effect on therapy compliance regarding the RT.

## Discussion

Interestingly, the results shown here refute the notion that smoking and co-morbidities, alone or in combination, negatively impact compliance with R(C)T in HNSCC patients. Whereas, increasing tobacco use is significantly associated with the presence of co-morbidities, there is no correlation between smoking and/or co-morbidities and an impaired achievement of R(C)T dosages. The vast majority (82.6%) of patients in the present study population does reach the required target dosage (200 mg/m^2^ BSA cisplatin) of their individual therapy regimen irrespective of smoking habit and/or co-morbidity. To this end, poor survival data of smokers in comparison to non-smokers cannot be explained by less intense treatment due to smoking associated co-morbidity. These data are in line with findings by Bøje et al. (17) who discouraged the assumption that co-morbidity affects treatment compliance. Moreover, cessation of therapy due to incompliance or treatment related morbidity which in general is a rare event in this study population (4.4% of the patients) occurs among smokers and patients with co-morbidities as often as among non-smokers and patients without co-morbidities (data not shown).

As expected, with increasing tobacco abuse smokers show increasing numbers of co-morbidities and the occurrence of such co-morbidity is age-dependent. It can be interpreted that co-morbidities sign responsible for earlier deaths and impaired outcome in tumor survival analysis in comparison to patients without co-morbidities. However, the latter seems independent of cancer disease and treatment. This finding is again in line with data from Bøje et al. stating that cancer specific death is not affected by co-morbidity suggesting that patients die from their co-morbidities rather than from their cancer (10, 17). In the present study, the proportion of active, former and never smokers is 54.3, 28, and 17.6%, respectively, with the latter being within the range of never smokers among comparable study populations (20–22). Roughly 80% of HNSCC patients, therefore, do have a positive smoking history, which holds true for populations outside and inside the USA with the latter often being associated with populations with rather low rates of smokers (20–22). In light of the eight edition of the TNM-classification (AJCC/UICC) the high proportion of smokers among HNSCC patients is important since, different from the HPV-status with positive impact on patient's survival in case of HPV infection, smoking with significant negative impact on patient's survival is not considered (23).

Only recently and investigating the same study population as analyzed here, we described that former smokers show survival rates as bad as active smokers (24). Table 2 and Figure 2 elucidate that the latter can only be attributed to those former smokers with co-morbidities since the former smokers without co-morbidities survive as well as never smokers without co-morbidities. Likewise, never and active smokers with co-morbidities show inferior survival rates in comparison to never and active smokers without co-morbidities. Therefore, in survival estimates the presence of co-morbidities is more important than the smoking habit itself. In a previous study investigating the influence of smoking on HPV-positive and HPV-negative TSCC we reported that the positive impact of HPV-positivity on survival is fully jeopardized by a positive smoking history (5). According to the results presented here, this, however, only might be true for those patients with co-morbidities since the negative effects of smoking are in particular evident in patients with co-morbidities in the present investigation. Vice versa, in the absence of any co-morbidity the effect of smoking appears to have only minor impact on survival. Since the occurrence of co-morbidities is age-dependent with never, former and active smokers being younger when not presenting with co-morbidities, additionally age seems to be less important in comparison to co-morbidities in survival estimates. Future investigations will clarify the latter further.

In the mentioned previous study investigating the same study population (24), we reported that alteration of smoking habit at time of first cancer diagnosis significantly (test for trend) improves survival, with no difference between quitting and reduction of the tobacco consumption. The latter, however, only could be observed for patients treated by surgery only. Taken this together with the results described here, it can be hypothesized that smoking and co-morbidities impact survival differently from what often is assumed: it is not the case that smoking induced co-morbidities jeopardize set treatment plans for R(C)T, but smoking itself seems to have a direct negative effect on specifically peri-operative features. This, however, can be counteracted if patients alter their smoking habit at time of cancer diagnosis. Co-morbidities, however, seem to influence survival rather independent from performed treatment.

Limitations of the study are based on the retrospective nature of the study design. Due to the latter, some questions could not be thoroughly answered such as (1) whether or not treatment planning by radiation oncologists initially was different for patients with a smoking habit and co-morbidities. Since, however, treatment dosage and substances of active and never smokers and patients with and without co-morbidities were the same in the present study, there only seems to be a small chance for a bias in this matter. The latter assumption is in line with data by Gourin et al. (25) investigating 75 patients. These authors could not find a correlation between severity of co-morbidities and choice of treatment. (2) When discussing the influence of co-morbidities, smoking related and unrelated, on survival in general and, moreover, in terms of endangering treatment dosage, prescribed medications for respective co-morbidities are rather neglected. However, the question should be addressed, what impact, for instance, high blood pressure may have on the patient's course of tumor disease when the blood pressure is neatly corrected by a sufficient anti-hypertensive medication. The assumed positive influence of co-morbidity associated medications weakening the negative impact of co-morbidities itself on the course of treatment of the patients could not be supported by detailed information on the prescribed medication of the study population since present data from patient files and tumor documentation were not uniform and not precise enough. Initially, the influence of sufficient vs. in-sufficient medication of co-morbidities on treatment and outcome should be part of the study design, since—mostly due to incompliance—there was a proportion of patients not being sufficiently treated for their co-morbidities. However, the number of patients without sufficient medication was not high enough to reach significance in subgroup analysis. (3) Even though data on alcohol consumption of the study population was collected, these data were excluded from analysis for two reasons: (i) both, smoking and drinking habit, rely on values of subjective impressions of the patients which specifically for drinking is not easy to specify in terms of defined measures. For smoking, however the applied measure “pack per day” is an easy to handle benchmark for patients and clinicians. (ii) different from “pack per day” there is no well-defined measurement for alcohol consumption or these measures are not easy to follow. Aarstad et al. (26), for instance, defined a regularly drinking person when the latter reported one drink more than twice a week. To in future overcome the defined limitations we initiated, in cooperation with the radiation oncologists of our head and neck tumor center, a prospective study.

## Conclusions

Smoking and co-morbidities, alone or in combination, do not negatively influence dose achievement of R(C)T in HNSCC patients. A positive smoking history and overall co-morbidity might impair the conduct of radio(chemo)therapy to a lesser extent than assumed. Smoking seems to have direct effects on peri-operative complications with consecutive impact on survival and, moreover, does cause co-morbidities. Co-morbidities, however, cause deaths which are independent from tumor treatment and treatment outcome. For survival estimates co-morbidity and age seems to be more important than the smoking habit of the patients.

## Data Availability Statement

The datasets generated for this study are available on request to the corresponding author.

## Ethics Statement

The study was approved by the Ethics Committee of the Medical Faculty of the Christian-Albrechts University of Kiel, Number D 507/17. Written informed consent was obtained from all participants.

## Author Contributions

MH, EQ, and KH: study design. TS, KK, AFab, AH, and MD: data acquisition. EQ and MH: statistical analysis. AFaz, ML, and MH: data interpretation. AFaz, AFab, EQ, and MH: manuscript preparation. AFab, KH, and AH: manuscript editing. All authors consented in publication of the manuscript.

### Conflict of Interest

The authors declare that the research was conducted in the absence of any commercial or financial relationships that could be construed as a potential conflict of interest.

## References

[1] Miller DL Puricelli MD Stack MS. Virology and molecular pathogenesis of HPV (human papillomavirus)-associated oropharyngeal squamous cell carcinoma. Biochem J. (2012) 443:339–53. 10.1042/BJ20112017 22452816 PMC3571652

[2] Marty R Roze S Bresse X Largeron N Smith-Palmer J. Estimating the clinical benefits of vaccinating boys and girls against HPV-related diseases in Europe. BMC Cancer. (2013) 13:10. 10.1186/1471-2407-13-10 23298365 PMC3561184

[3] Marur S Forastier AA. Head and neck squamous cell carcinoma: update on epidemiology, diagnosis, and treatment. Mayo Clin Proc. (2016) 91:386–96. 10.1016/j.mayocp.2015.12.017 26944243

[4] Ang KK Harris J Wheeler R Weber R Rosenthal DI Nguyen-Tân PF. Human papillomavirus and survival of patients with oropharyngeal cancer. N Engl J Med. (2010) 363:24–35. 10.1056/NEJMoa0912217 20530316 PMC2943767

[5] Hoffmann M Quabius ES Tribius S Gebhardt S Görögh T Hedderich J. Influence of HPV-status on survival of patients with tonsillar carcinomas (TSCC) treated by CO_2_ surgery plus risk adapted therapy - a 10 year retrospective single centre study. Cancer Lett. (2018) 413:59–68. 10.1016/j.canlet.2017.10.045 29100961

[6] Anantharaman D Abedi-Ardekani B Beachler DC Gheit T Olshan AF Wisniewski K. Geographic heterogeneity in the prevalence of human papillomavirus in head and neck cancer. Int J Cancer. (2017) 140:1968–75. 10.1002/ijc.30608 28108990 PMC8969079

[7] Fakhry C1 Gillison ML. Clinical implications of human papillomavirus in head and neck cancers. J Clin Oncol. (2006) 24:2606–11. 10.1200/JCO.2006.06.1291 16763272 PMC4696042

[8] Ahmadi N Chan M Huo YR Sritharan N Chin RY. Survival outcome of tonsillar squamous cell carcinoma (TSCC) in the context of human papillomavirus (HPV): a systematic review and meta-analysis. Surgeon. (2019) 17:6–14. 10.1016/j.surge.2018.04.009 29843958

[9] Sharp L McDevitt J Carsin AE Brown C Comber H. Smoking at diagnosis is an independent prognostic factor for cancer-specific survival in head and neck cancer: findings from a large, population-based study. Cancer Epidemiol Biomarkers Prev. (2014) 23:2579–90. 10.1158/1055-9965.EPI-14-0311 25128401

[10] Bøje CR. Impact of comorbidity on treatment outcome in head and neck squamous cell carcinoma-a systematic review. Radiother Oncol. (2014) 110:81–90. 10.1016/j.radonc.2013.07.005 23953753

[11] Beynon RA Lang S Schimansky S Penfold CM Waylen A Thomas SJ. Tobacco smoking and alcohol drinking at diagnosis of head and neck cancer all-cause mortality: results from head and neck 5000, a prospective observational cohort of people with head and neck cancer. Int J Cancer. (2018) 143:1114–27. 10.1002/ijc.31416 29607493 PMC6099366

[12] Peterson LA Bellile EL Wolf GT Virani S Shuman AG Taylor JM University of Michigan Head and Neck Specialized Program of Research Excellence Program. Cigarette use, comorbidities, and prognosis in prospective head and neck squamous cell carcinoma population. Head Neck. (2016) 38:1810–20. 10.1002/hed.24515 27432208 PMC5391974

[13] McCarter K Baker AL Britton B Wolfenden L Wratten C Bauer J. Smoking, drinking, and depression: comorbidity in head and neck cancer patients undergoing radiotherapy. Cancer Med. (2018) 7:2382–90. 10.1002/cam4.1497 29671955 PMC6010893

[14] Haigentz M Jr Suarez C Strojan P Rodrigo JP Rinaldo A Bradford CR. Understanding interactions of smoking on prognosis of HPV-associated oropharyngeal cancers. Adv Ther. (2018) 35:255–60. 10.1007/s12325-018-0682-4 29511982

[15] Chen PC Yang CC Wu CJ Liu WS Huang WL Lee CC Factors predict prolonged wait time and longer duration of radiotherapy in patients with nasopharyngeal carcinoma: a multilevel analysis. PLoS ONE. (2014) 14:e109930 10.1371/journal.pone.0109930 PMC4196956 25314009

[16] Lee L Cheung WY Atkinson E Krzyzanowska MK Impact of comorbidity on chemotherapy use and outcomes in solid tumors: a systemic review. J Clin Oncol. (2011) 29:106–17. 10.1200/JCO.2010.31.3049 21098314

[17] Bøje CR Dalton SO Grønberg TK Primdahl H Kristensen CA Andersen E. The impact of comorbidity on outcome in 12 623 Danish head and neck cancer patients: a population based study from the DAHANCA database. Acta Oncol. (2013) 52:285–93. 10.3109/0284186X.2012.742964 23320773

[18] Charlson ME Pompei P Ales KL MacKenzie CR. A new method of classifying prognostic comorbidity in longitudinal studies: development and validation. J Chronic Dis. (1987) 40:373–83 10.1016/0021-9681(87)90171-8 3558716

[19] Quan H Li B Couris CM Fushimi K Graham P Hider P. Updating and validating the Charlson comorbidity index and score for risk adjustment in hospital discharge abstracts using data from 6 countries. Am J Epidemiol. (2011) 173:676–82. 10.1093/aje/kwq433 21330339

[20] Skillington SA Kallogjeri D Lewis JS Jr Piccirillo JF. Prognostic importance of comorbidity and the association between comorbidity and p16 in oropharyngeal squamous cell carcinoma. JAMA Otolaryngol Head Neck Surg. (2016) 142:568–75. 10.1001/jamaoto.2016.0347 27077485 PMC5085064

[21] Fakhry C Westra WH Wang SJ van Zante A Zhang Y Rettig E. The prognostic role of sex, race, and human papillomavirus in oropharyngeal and nonoropharyngeal head and neck squamous cell cancer. Cancer. (2017) 123:1566–75. 10.1002/cncr.30353 28241096 PMC5788020

[22] Habbous S Harland LT La Delfa A Fadhel E Xu W Liu FF Comorbidity and prognosis in head and neck cancers: differences by subsites, stage, and human papillomavirus status. Head Neck. (2014) 36:802–10. 10.1002/hed.23360 23616414

[23] Mahul BA Gress DM Meyer VLR Edge SB Greene FL Byrd DR, eds. AJCC Cancer Staging Manual. 8th ed New York, NY: Springer (2017).

[24] Fazel A Quabius ES Gonzales-Donate M Laudien M Herzog A Kress K Alteration of smoking habits with diagnosis influences survival of HNSCC patients. Nicotine Tobacco Res. (2019). 10.3892/mco.2020.2120 PMC7453390 32874580

[25] Gourin CG McAfee WJ Neyman KM Howington JW Podolsky RH Terris DJ. Effect of comorbidity on quality of life and treatment selection in patients with squamous cell carcinoma of the head and neck. Laryngoscope. (2005) 115:1371–75. 10.1097/01.mlg.0000167983.32017.64 16094107

[26] Aarstad HJ Osterhus AA Aarstad HH Lybak S Aarstad AKH General health-related quality of life scores from head and neck squamous cell carcinoma patients obtained throughout the first year following diagnosis predicted up to 10-year overall survival. Eur Arch Otorhinolaryngol. (2018) 257:207–17. 10.1007/s00405-017-4800-8 29159750

